# Supplementary material for: From Nature to Synthetic Compounds: Novel 1(N),2,3 Trisubstituted-5-oxopyrrolidines Targeting Multiple Myeloma Cells
Source: Int J Mol Sci. 2022 Oct 27;23(21):13061. doi: 10.3390/ijms232113061 (PMC9658545; doi:10.3390/ijms232113061)

Supplementary Material

Standard screening protocol of mobile phase

The standard screening protocol of mobile phase composition used for the first studies on enantioseparation of trans-12 is reported in Supplementary Table S1.

Mobile Phase Composition			
Entry	n-Hexane	2-propanol	Ethanol
1	90	10	
2	50	50	
3		100	
4			100
5	90		10

Supplementary Table S1. Standard screening protocol: mobile phase composition.

Analytical Screening

Results of the analytical screening are reported in Tables 2 and are expressed as retention (k), selectivity (α) and resolution (Rs) factors.

CSPs								
	Chiralpak IA[b,c]				Chiralpak IC[c]			
**Eluent[a]**	**k1**	**k2**	**α**	**RS**	**k1**	**k2**	**α**	**RS**
**A**	2.42	2.70	1.11	1.37	4.64	17.01	3.68	19.5
**B**	1.35	1.52	1.11	1.13	2.68	8.49	3.17	15.6
**C**	0.97	1.09	1.12	0.77	1.92	6.07	3.16	13.4
**D**	0.72	0.78	1.09	-	1.39	4.17	3.00	10.6
**E**	0.57	0.61	1.06	-	1.10	3.15	2.87	10.0
**F**	0.31	-	-	-	0.62	1.52	2.45	5.49
**G[d]**	0.24	-	-	-	0.25	0.49	1.96	1.46
**H[d]**	0.23	-	-	-	0.28	-	-	-
**I**	1.02	-	-	-	1.09	1.77	1.63	3.02
**L**	1.52	-	-	-	1.48	2.56	1.73	3.44
**M**	2.35	2.46	1.05	-	2.41	4.39	1.82	7.37

Supplementary Table S2. [a]Eluent composition: A, n-Hex/ IPA (90:10 v/v); B, n-Hex/IPA (85:15 v/v); C, n-Hex/IPA (80:20 v/v); D, n-Hex/IPA (75:25 v/v); E, n-Hex/IPA (70:30 v/v); F, n-Hex/IPA (50:50 v/v); G, IPA (100); H, EtOH (100); I, n-Hex/EtOH (80:20 v/v); L, n-Hex/EtOH (85:15 v/v); M, n-Hex/EtOH (90:10 v/v); [b] Mobile phase was added with 0.1% DEA; [c] Mobile phase was added with 0.3% TFA; Flow rate: 1.0 mL/min; [d] Flow rate: 0.5 mL/min; Concentration: 1 mg/mL; Injection volume: 10 µL; detection at 220 nm.

Biological investigation

RPMI 8226 cells were treated with different concentrations (10, 100 and 500 µM) of each compound and, after 24 hours, proteasome activity assay was performed.

Supplementary Figure S1. Proteasome activity of RPMI 8226 cells treated with (2R,3R/2S,3S)-13, 14, 16 and 20. Proteasome activity was evaluated in cells treated for 24 hours with different concentrations (10, 100 and 500 µM) of compounds. The graphs represent the mean proteasome activity ± SD, compared to untreated controls, arbitrarily set to 100%. ** p < 0.01 vs CTRL.

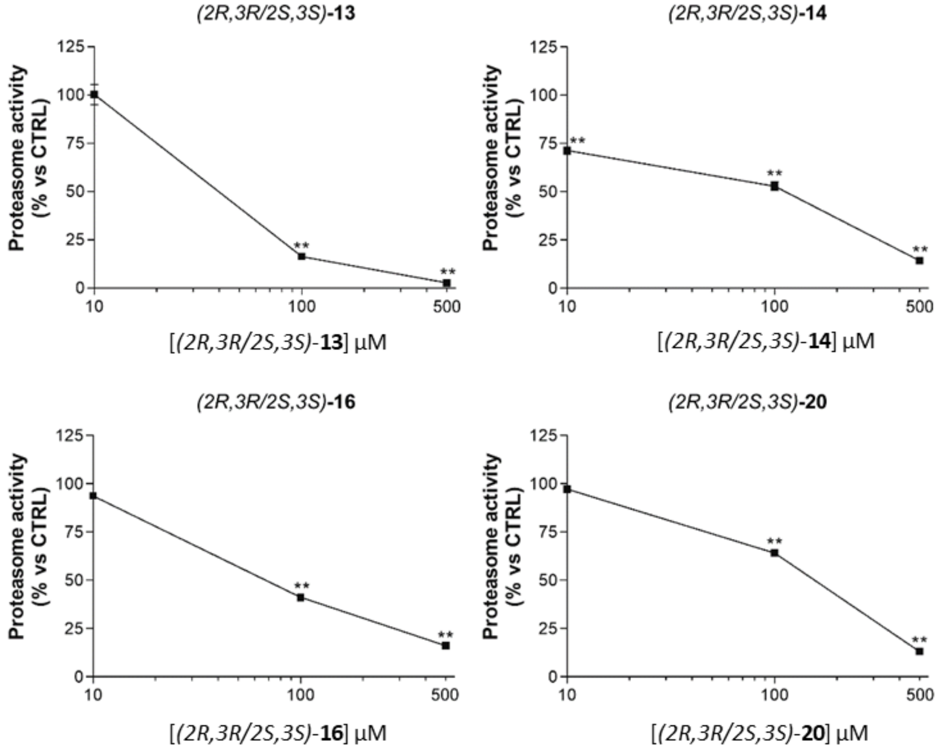

RPMI 8226 cells treated with BTZ 1, 5 and 10 nM were used as positive control for all experiments.

Supplementary Figure S2. A) MTT assay of BTZ in RPMI 8226 cells. Cells were not treated (CTRL) or treated with different concentrations of BTZ (1, 5 and 10 nM) for 24, 48 and 72 hours. The graphs represent the mean percentage ± SD of cell viability, compared to untreated controls, arbitrarily set to 100%. ** p < 0.01 vs CTRL; B) Trypan blue vital count assay of RPMI 8226 cells treated with BTZ. Cells were not treated (CTRL) or treated with different concentrations of compounds (1, 5 and 10 nM) for 24 hours. The graphs represent the mean cell number ± SD of viable and dead cells. ** p < 0.01 vs CTRL; C) Proteasome activity of RPMI 8226 cells treated with BTZ. Proteasome activity was evaluated in cells treated for 24 hours with different concentrations (1, 5 and 10 nM) of BTZ. The graphs represent the mean proteasome activity ± SD, compared to untreated controls, arbitrarily set to 100%. ** p < 0.01 vs CTRL.

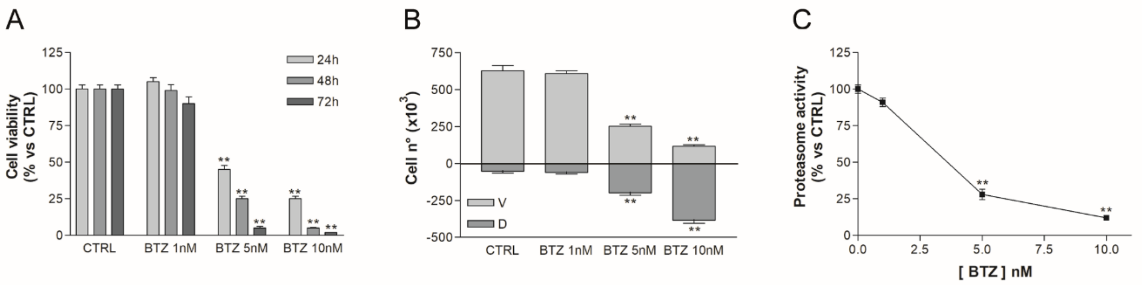

Supplement: Supplementary file 1 [file ijms-23-13061-s001.zip › ijms-1979115-supplementary.pdf]
